# Thoughts Around the Zika Virus Crisis

**DOI:** 10.1007/s11908-016-0551-8

**Published:** 2016-11-10

**Authors:** Didier Musso, Marion C. Lanteri

**Affiliations:** 1 Institut Louis Malardé, PO Box 30, 98713 Papeete, Tahiti French Polynesia; 2 Blood Systems Research Institute, 270 Masonic Ave., San Francisco, CA 94118 USA

**Keywords:** Zika virus, outbreak, emergence, crisis

## Abstract

As a lot of reviews are available on Zika virus (ZIKV), in this short commentary, we will focus on the recent advances and gaps in knowledge regarding our understanding of ZIKV infections and on the reaction to the “ZIKV crisis.”

## Recent Advances and Gaps in Knowledge

Over the 60 years following ZIKV discovery and prior to the ZIKV outbreak on the Yap Island in 2007, less than 20 human ZIKV infections had been confirmed. The first severe neurologic complications associated with ZIKV infection (Guillain-Barré syndrome (GBS)) and the potential for materno-fetal, sexual, and post-transfusion transmission were first described during the 2013–2014 French Polynesian outbreak. Even though they were published in high impact factor reviews, data from French Polynesia were not really taken into consideration. The emergence of ZIKV in the Americas in 2015 was associated with a dramatic increase in incidence of microcephaly. As of August 2016, ZIKV is continuing its spread in the Americas with the first autochthonous infections reported in the contiguous continental US in Florida. While ZIKV is still circulating in the Pacific, ZIKV outbreaks were recently reported in Africa and Southeast Asia. There is a high potential for ZIKV to spread in sub-Saharan Africa and Southeast Asia as well as on the Indian Ocean Islands.

Nevertheless, the real burden of ZIKV is unknown. In the past, ZIKV infections have been underreported because infections were misdiagnosed and thought to be caused by other related arboviruses such as dengue virus (DENV) and laboratory diagnostic tools were lacking. In countries like Vietnam, Thailand, Maldives, and Vanuatu, ZIKV circulation has been confirmed only by the detection of imported cases (travelers returning from the area and developing symptoms were further investigated and diagnosed as ZIKV related) proving that ZIKV was circulating in these regions but was undetected. On the other hand, the number of infections actually reported relies only on estimates and suspected cases, over or under estimating the number of infections.

ZIKV, like DENV, adapted from feral mosquitoes and monkeys in a sylvatic zoonotic transmission cycle to *Aedes aegypti* and *Aedes albopictus* as vectors, and to humans as reservoir in an urban transmission cycle, making the 2.2 billion people leaving in ZIKV-compatible environment at risk for ZIKV infection. To date, ZIKV does not seem to have adapted to *Culex* sp. mosquitoes and therefore the main vector for West Nile virus (WNV) is not a competent vector for ZIKV. Recent findings support vertical transmission of ZIKV in *Ae. aegypti*, as demonstrated in the past for DENV and yellow fever virus (YFV), providing a mechanism for persistence during adverse climatic conditions or in the absence of susceptible vertebrate hosts. New mosquito vector control strategies such as release of *Wolbachia* infected, genetically modified or irradiated mosquitoes are promising, but large scale implementation remains challenging.

Sexual transmission from man to woman, man to man, and recently woman to man has been reported. The impact of ZIKV sexual transmission in active ZIKV areas is impossible to evaluate because all the population is also exposed to mosquito bites. The maximum duration of infectivity in semen is unknown.

If the link between ZIKV and microcephaly is established, the number of microcephaly cases related to ZIKV is unknown because all reported cases are not confirmed and all etiologies of microcephaly are not investigated. For example, as of June 2016, Brazil reported 8165 cases of microcephaly and other central nervous system abnormalities; of these, 1638 were confirmed cases of microcephaly including 270 laboratory confirmed cases of ZIKV-related microcephaly. In vitro studies using neural progenitor cells and animal models have demonstrated the neurotropism of ZIKV.

Gaps on materno-fetal complications of ZIKV have been recently reviewed in the Lancet: “We know that a pregnant woman infected with ZIKV runs a risk of having a microcephalic baby but we can’t tell her how high that risk is and how it might evolve over the course of her pregnancy. We don’t know what is the full spectrum of the ZIKV-caused congenital defects. Will apparently unaffected children whose mothers had ZIKV in pregnancy develop normally? Will they be able to walk and talk normally? Will they be mentally impaired or have other problems that only become evident years later?”

Within the *Flavivirus* genus, there is an immunity against homologous flaviviruses. Immunity against DENV is not protective against ZIKV, as demonstrated by the emergence of ZIKV in high endemic areas for DENV. On the contrary, in vitro studies suggest that previous immunity to DENV may increase the risk for severe ZIKV infection but this needs further investigation in large longitudinal human cohorts. The duration of immunity against ZIKV is unknown.

The two main ZIKV lineages are African and Asian. To date, all complications are associated with the Asian lineage and, within the Asian lineage, only since ZIKV emerged in French Polynesia (no complication described during the first ZIKV outbreak in Yap, 2007, also causes by the Asian lineage). In vitro studies suggest a cross-protection against ZIKV Asian lineage by antibodies against ZIKV African lineage.

The main favorable predisposing factor of ZIKV emergence is probably its adaptation to new mosquito vectors. Mutations associated with increased virulence have been described for other arboviruses such as CHIKV and WNV, even if genetic changes are detected in the currently circulating strains of ZIKV, the correlation between mutations and increased virulence has not been demonstrated. Other favorable factors for emergence are increased urbanization, tourism industry, and travel exchanges as well as lack of effective mosquito control and viral introduction in areas lacking herd immunity against ZIKV.

Diagnosis of ZIKV infections relies on molecular diagnosis and/or seroneutralization tests which can only be performed by specialized laboratories and with no rapid test currently available, ZIKV diagnosis is a challenge in resource limited countries.

## Reacting to the ZIKV Crisis

ZIKV emergence is dramatic for many countries and preventive measures are needed in active ZIKV transmission areas; however, in areas lacking ZIKV competent mosquito vectors, the risk for ZIKV emergence is minimal and ZIKV-related concerns should be limited to pregnant women who might have contracted the infection during travels to active ZIKV transmission areas or have been contaminated by infected partners.

Sexual transmission of ZIKV is in the spotlight but it should be kept in mind that ZIKV is principally a mosquito-borne pathogen. Even if ZIKV can be transmitted by sexual intercourse, it is far from being the most dangerous sexual transmitted pathogen.

Recently, ZIKV emerged on the continental US in the state of Florida and few cases of ZIKV transmission by blood transfusion have been reported in Brazil. Health authorities requested countrywide implementation of ZIKV nucleic acid testing (NAT) for the screening of all individual blood donations in the USA, even in states without competent mosquito vectors. On the other hand, in several African countries, only insensitive rapid tests are performed to screen blood donors for HIV. Pathogen inactivation of blood products, when possible, could represent an interesting alternative to NAT blood screening, especially in at-risk areas where mosquito vectors are present and for blood components to be transfused to high risk recipients (pregnant women).

Another exaggerated reaction was the recommendation to cancel the Olympic Games in Brazil while the local entomologists and infectiologists agreed that in August, the risk for ZIKV transmission would be low in Brazil. Indeed, in Brazil, the arbovirus season is from January to April. In addition, the Olympic games attracted a limited number of foreigners outside of the participating athletes and the accompanying staff and represent a minor population compared to the daily flow of international travelers making the trip for professional or touristic reasons.

Another questionable move was that because of lack of funds dedicated to ZIKV, the redirection of funds that were allocated to other causes such as Ebola, Malaria, YFV to support urgently needed research. A tribune has been published in Nature in September 2016 entitled “the US government should not redirect vital funds to work on ZIKV at the expense of other health priorities,” like yellow fever or malaria that threaten Latin America and Africa. Indeed, there is an urgent need to fill in the gap of knowledge to inform adequate reaction to ZIKV but one needs to keep in mind that humanity is under constant threat and the risk for other infectious agents to emerge and become the next most frightening agent is high. Therefore, instead of redirecting funds attributed to important research, additional funding should be released to face the threat posed by ZIKV.

## Conclusion

Since its emergence in the USA, ZIKV has captured the attention from the international community for the past year. The future of ZIKV is unpredictable but the story of ZIKV is certainly not finished. Nevertheless, other pathogens like malaria, HIV, and Ebola should not be neglected, and efforts for other arboviruses such as DENV which is a concern for billions of children and adults throughout the tropics and YFV for which a vaccine is available should continue.

